# Self-Healing Fire Prevention and Extinguishing Hydrogel Derived from Carboxymethyl Cellulose-Modified Amphiphilic Copolymers

**DOI:** 10.3390/gels11110901

**Published:** 2025-11-10

**Authors:** Lingyu Ge, Bin Xu

**Affiliations:** 1 College of Chemical Engineering and Materials, Shandong University of Aeronautics, Binzhou 256600, China; gly726645752@126.com; 2 Shandong Key Laboratory of Eco-Environmental Science for the Yellow River Delta, Shandong University of Aeronautics, Binzhou 256600, China

**Keywords:** carboxymethyl cellulose-modified amphiphilic polymer, self-healing fire prevention and extinguishing hydrogel, coal spontaneous combustion, viscoelasticity, thermal stability

## Abstract

Gel materials are widely used in underground mining for air leakage sealing and coal spontaneous combustion prevention. In this study, a novel self-healing carboxymethyl cellulose-modified amphiphilic polymer hydrogel with fire prevention and extinguishing capabilities is synthesized through ionic crosslinking between CMC-*graft*-poly(AM-*co*-NaA-*co*-BAM) and aluminum citrate (AlCit). The copolymer is constructed by grafting sodium carboxymethyl cellulose (CMC) onto an amphiphilic polymer backbone composed of acrylamide (AM), sodium acrylate (NaA), and N-benzylacrylamide (BAM), forming a dual-network structure via hydrophobic association and hydrogen bonding. The carboxymethyl cellulose-modified amphiphilic polymer demonstrates optimal viscosity-enhancing performance at a CMC content of 7.5 wt%. CMC-*graft*-poly(AM-*co*-NaA-*co*-BAM) demonstrated superior temperature, shear, and salt resistant performance compared with poly(AM-*co*-NaA-*co*-BAM), poly(AM-*co*-NaA), and CMC polymers, as well as enhanced viscoelasticity and self-healing capability. When crosslinked with AlCit, CMC-*graft*-poly(AM-*co*-NaA-*co*-BAM)-AlCit gel demonstrated superior viscoelastic properties and self-healing capability, as well as thermal stability, which gave the superior fire prevention and extinguishing performance for charcoal in fire extinction tests. CMC-*graft*-poly(AM-*co*-NaA-*co*-BAM) has abundant cross-linking sites, which lead to accelerated gelation and improved mechanical strength, while the hydrophobic microdomains acted as physical cross-linking points that interconnected polymer chains into a three-dimensional network. The hydrophobic interactions within the hydrogel are dynamically reversible. This intrinsic property allows physical cross-links to spontaneously reassociate when fracture surfaces make contact. Consequently, the material exhibits autonomous self-healing.

## 1. Introduction

China’s energy resource endowment is characterized by “abundant coal, scarce oil, and limited natural gas,” which makes coal the cornerstone of national energy security [1]. For the foreseeable future, coal will remain the dominant energy source in China. However, coal mining is plagued by coal fire disasters, especially with increasing mining depths and the advancement of fully mechanized mining technologies; the risk of coal spontaneous combustion becomes more severe [2]. Preventing and controlling coal fires is critical to ensure safe coal extraction [3].

In the field of fire prevention and suppression, materials and technologies have matured significantly, with commonly employed techniques including inert gases [4,5,6], three-phase foam [7,8,9], inhibitors [10,11], sealing agents [12], and hydrogels [13,14].

Gel materials are widely used in underground mining for air leakage sealing and coal spontaneous combustion prevention. However, conventional inorganic silica gels, such as water glass-based gels [15,16,17], have disadvantages such as poor elasticity, high brittleness, and a tendency to crack or undergo plastic deformation, significantly reducing their effectiveness in air leakage blockage and fire suppression. Although traditional organic hydrogels exhibit improved performance, their mechanical properties remain relatively weak [18]. Therefore, the design and research of gels with strong mechanical properties have attracted extensive attention [19,20].

Hydrogel is a “soft material” that rapidly swells in water to reach equilibrium while maintaining its shape and three-dimensional network structure [21]. With excellent viscoelasticity and biocompatibility, it has attracted widespread attention in fire prevention and suppression applications [14,22,23]. Particularly in coal fire prevention and control, polyacrylamide (PAM)-based hydrogels have emerged as the most prominent solution [24]. PAM’s amide-rich linear backbone enables versatile chemical modification for synthesizing derivatives and tailored hydrogels [25,26,27]. Through chemical modification, various PAM derivatives can be synthesized, enabling the development of functionally modified PAM hydrogels. However, the relatively poor mechanical properties and brittleness of PAM hydrogels have limited their practical application in fire prevention scenarios [28].

To address these mechanical limitations, researchers have developed advanced hydrogel systems by incorporating various energy dissipation mechanisms, dynamic interactions, or novel cross-linking agents such as hyperbranched polycaprolactone. These innovations have led to hydrogels with superior mechanical performance [29,30], including double-network hydrogels and hydrophobic association hydrogels [31,32,33], which show great promise for fire prevention applications [34].

In recent years, degradable natural polymers such as cellulose have been widely utilized in the construction of gel systems due to their advantages of low raw material cost [24,35,36], processability, biocompatibility, and biodegradability [37,38]. Physical cross-linking mechanisms include hydrogen bonding and hydrophobic interactions, while chemical cross-linking approaches encompass free radical copolymerization and dynamic covalent bonding. Most cellulose-based hydrogels are soft and brittle, prompting extensive research into the design of elastic hydrogels [39]. The graft copolymerization of cellulose or its derivatives with water-soluble monomers is recognized as an effective method to introduce ionizable or other polar groups into the cellulose backbone [40,41]. For instance, cross-linked polyacrylamide chains can be grafted onto carboxymethyl cellulose (CMC) via free radical polymerization using cross-linking agents such as MBA or epichlorohydrin (EPI) [42,43]. Alternatively, sodium carboxymethyl cellulose can serve as the primary component, combined with suitable cross-linking agents, to synthesize cellulose-based hydrogels [44]. Both strategies enable the preparation of highly absorbent hydrogels [45]. Zhou et al. [46] developed a novel self-healing biomass hydrogel material using sodium carboxymethyl cellulose (CMC) as the primary agent, iron-aluminum citrate (Fe-AlCit) as a cross-linker, and glucono-δ-lactone (GDL) as a pH regulator. Fourier transform infrared (FTIR) spectroscopy and other microstructural characterization methods confirmed that the hydrogel exhibits abundant hydrogen bonds and ionic bonds. Driven by these interactions, the hydrogel demonstrated rapid self-repair capability, restoring its damaged network to the pre-cut state within 90 s after being bisected. Temperature-programmed oxidation (TPO) experiments revealed that coal samples treated with this hydrogel achieved CO and C_2_H_4_ inhibition rates of 44.84% and 46.57%, respectively, at 220 °C. Infrared spectral analysis further demonstrated that the hydrogel reduced the content of -OH and Ar-C-O groups in coal by 49.58% and 40.82%, respectively, while increasing stable C=C content in aromatic hydrocarbons by 6.14%. These modifications effectively disrupted the coal-oxygen chain reaction, significantly suppressing spontaneous combustion of coal. The rheological behavior of N-benzylacrylamide (BAM)-modified partially hydrolyzed polyacrylamide (HPAM) was studied by Jiang et al. [47] and the results showed that the phenyl group of BAM, owing to its moderate size and weak self-association, preferentially facilitates the formation of dynamic mixed micelles, thereby avoiding intense intramolecular aggregation, while its rapid micellar kinetics enable reversible transitions, enhancing shear adaptability and self-healing capability.

In this study, a carboxymethyl cellulose-modified amphiphilic polymer was prepared by introducing an amphiphilic polymer into the molecular chain of a natural polymer to enhance the structural strength and stabilize the spatial network of the polymer, and then gels were synthesized by crosslinking the cellulose-modified amphiphilic polymers with aluminum citrate. The properties and microstructure of the gel were characterized through various tests, and its fire extinguishing performance was evaluated via small-scale fire suppression experiments. By incorporating cellulose modification, a unique dual-network structure was introduced, combining hydrophobic association and hydrogen bonding interactions to construct a stable three-dimensional crosslinked network. This design endowed the hydrogel with excellent mechanical strength and dynamic self-healing capability—where hydrophobic microdomains acted as physical crosslinking points, granting the material autonomous self-healing properties.

## 2. Results and Discussion

### 2.1. Viscosity-Enhancing Properties of Different Copolymers

**Figure 1:**
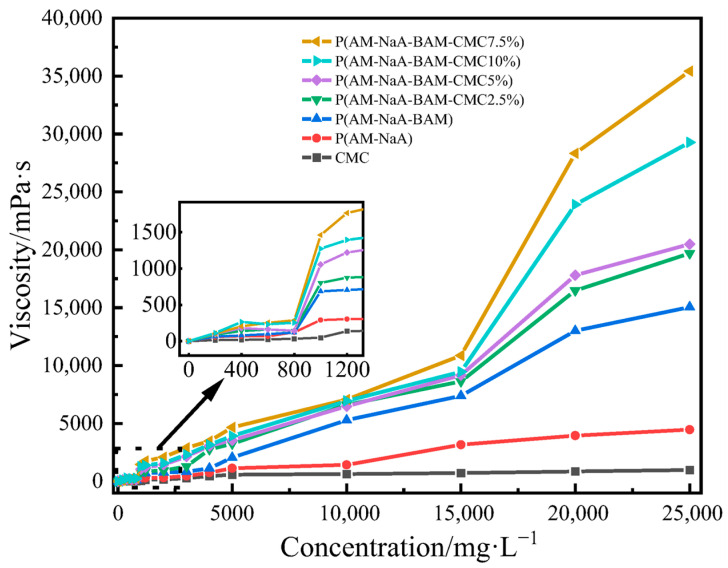
Viscosity of different copolymers solutions.

The influence of carboxymethyl cellulose content on the apparent viscosity of the copolymer solutions was evaluated, and the results are shown in Figure 1. The results demonstrated that CMC-*graft*-poly(AM-*co*-NaA-*co*-BAM) exhibited superior viscosity-enhancing performance compared with poly(AM-*co*-NaA-*co*-BAM), poly(AM-*co*-NaA), and CMC. This enhanced performance can be attributed to the structural limitations of pure CMC, which relies solely on hydrogen bonding through hydrophilic groups and lacks the three-dimensional network structure formed by hydrophobic association. CMC7.5%-*graft*-poly(AM-*co*-NaA-*co*-BAM) exhibited superior viscosity-enhancing performance probably due to the following dual synergistic mechanisms: (1) molecular chain extension induced through electrostatic repulsion of carboxylate anions from sodium acrylate was utilized to increase entanglement density for effective dissipation of thermal stress, thereby inhibiting viscosity decay under elevated temperatures and (2) a comb-like topological structure collaboratively constructed by grafted acrylamide side chains and the backbone of carboxymethyl cellulose (CMC) was engineered to strengthen interchain physical entanglements while optimizing energy dissipation capability under thermal loading conditions through its multistage architecture.

### 2.2. Thermal and Salt Resistance Performance of Different Copolymers

**Figure 2:**
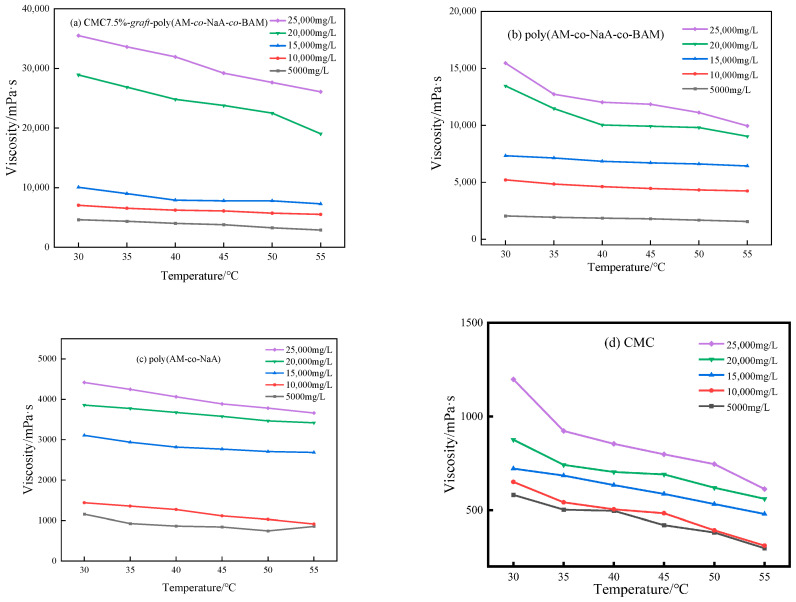
Thermal resistance performance of CMC7.5%-*graft*-poly(AM-*co*-NaA-*co*-BAM), poly(AM-*co*-NaA-*co*-BAM), poly(AM-*co*-NaA), and CMC.

**Figure 3:**
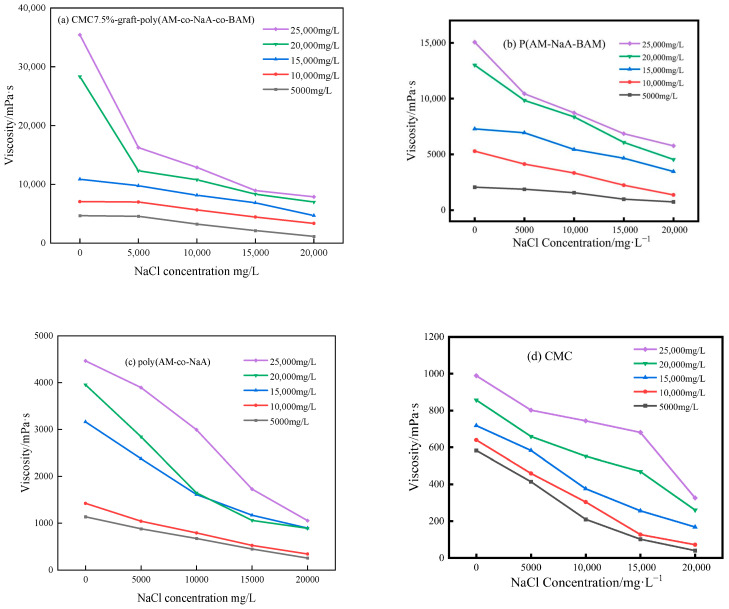
Salt resistance performance of CMC7.5%-*graft*-poly(AM-*co*-NaA-*co*-BAM), poly(AM-*co*-NaA-*co*-BAM), poly(AM-*co*-NaA), and CMC under varying NaCl concentrations.

**Figure 4:**
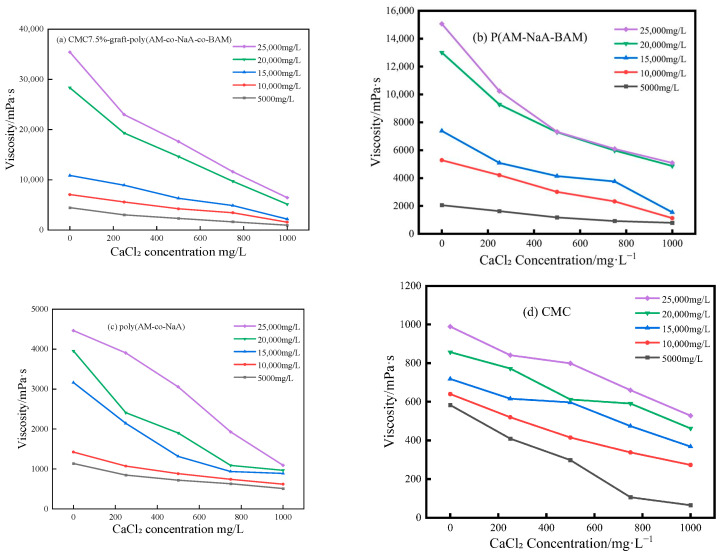
Salt resistance performance of CMC7.5%-*graft*-poly(AM-*co*-NaA-*co*-BAM), poly(AM-*co*-NaA-*co*-BAM), poly(AM-*co*-NaA) and CMC under varying CaCl_2_ concentrations.

Thermal and salt resistance performance of copolymer solutions are shown in Figure 2, Figure 3 and Figure 4. CMC7.5%-*graft*-poly(AM-*co*-NaA-*co*-BAM) showed superior and significant thermal and salt resistance performance compared with poly(AM-*co*-NaA-*co*-BAM), poly(AM-*co*-NaA), and CMC, primarily attributed to its molecular structure.

The enhanced temperature and salt tolerance of the CMC-*graft*-poly(AM-*co*-NaA-*co*-BAM) copolymers is attributed to the synergistic interaction of its functional groups and a dynamic reversible cross-linking mechanism. Under elevated temperatures (30–55 °C), the thermal motion of molecular chains is suppressed by the rigid ring structure and interchain hydrogen-bonding network of the carboxymethyl cellulose (CMC) skeleton, while the hydrophobic microdomains formed by the N-phenylacrylamide (BAM) monomer are strengthened in their association through a dynamic dissociation–recombination process, thereby dissipating thermal energy and preserving the integrity of the network structure, as evidenced by its significantly higher viscosity retention rate compared to reference samples in Figure 2. With regard to salt tolerance, the extended chain conformation is maintained by the strong electrostatic repulsion generated by the anionic groups (-COO^−^) from the ionization of sodium acrylate (NaA) units, whereas the shielding effect of salt ions on charged groups is physically obstructed by the microdomains formed by the BAM hydrophobic units, resulting in stable hydrodynamic volume and viscosity across different concentrations of NaCl and CaCl_2_ solutions, as illustrated in Figure 3 and Figure 4, where salt resistance is markedly superior to that of the control group.

### 2.3. Copolymers Characterization

#### 2.3.1. Infrared Spectroscopic Analysis

**Figure 5:**
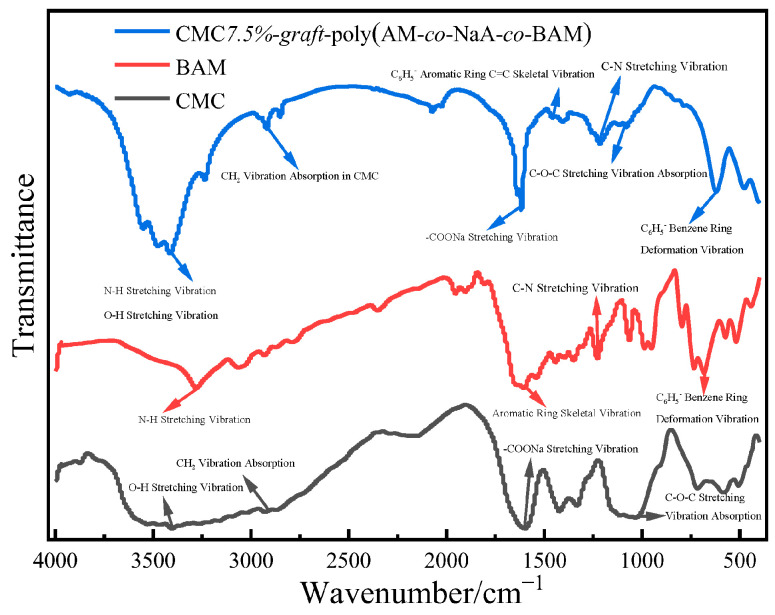
The FT-IR spectra of CMC7.5%-*graft*-poly(AM-*co*-NaA-*co*-BAM), BAM, and CMC.

The FT-IR spectrum of CMC-*graft*-poly(AM-*co*-NaA-*co*-BAM) (Figure 5) exhibited characteristic absorption bands corresponding to its molecular structure. A stretching vibration band attributed to both the amide group (-NH_2_) and the hydroxyl group (-OH) was observed at 3403.9 cm^−1^, while the -CH_2_ vibration from CMC appeared at 2933.9 cm^−1^. The asymmetric stretching vibration of -COONa was detected at 1610.7 cm^−1^. Aromatic structural features were confirmed by the C=C skeletal vibration at 1472.6 cm^−1^ and the benzene ring deformation vibration at 728.0 cm^−1^. Additional characteristic peaks included the C-N stretching vibration at 1259.0 cm^−1^ and the C-O-C stretching vibration at 1033.2 cm^−1^. These spectroscopic features collectively demonstrate the successful synthesis of the target copolymers.

#### 2.3.2. Relative Molecular Mass Analysis

The relative molecular weight of the copolymers was determined via the intrinsic viscosity method. Due to the self-assembled aggregate structures formed by hydrophobic monomers in amphiphilic copolymers, measurement deviations may occur. To mitigate this, β-cyclodextrin (β-CD) was employed to disrupt the associative structures by encapsulating hydrophobic groups, thereby extending the copolymer chains for accurate measurement [48], and cellulose-modified amphiphilic polymers were determined via the intrinsic viscosity method, in accordance with the Chinese national standard GB/T 12005.1-1989 (Determination of Intrinsic Viscosity of Polyacrylamide) [49] in Table 1.

CMC7.5%-*graft*-poly(AM-*co*-NaA-*co*-BAM) demonstrated the highest relative molecular mass among the compared systems, which is considered one of the primary reasons for its optimal viscosity-enhancing performance. The possible reason is that it has elongated molecular chains and more densely entangled structures, resulting in stronger intermolecular interactions, while a higher molecular weight necessitates greater energy input to disrupt the interactions between molecular chains or to induce relative motion within the entangled networks, while also enabling more effective resistance against the penetration and erosion of chemical substances.

### 2.4. Viscoelastic Test of Copolymers

#### 2.4.1. Viscoelasticity Measurements

**Figure 6:**
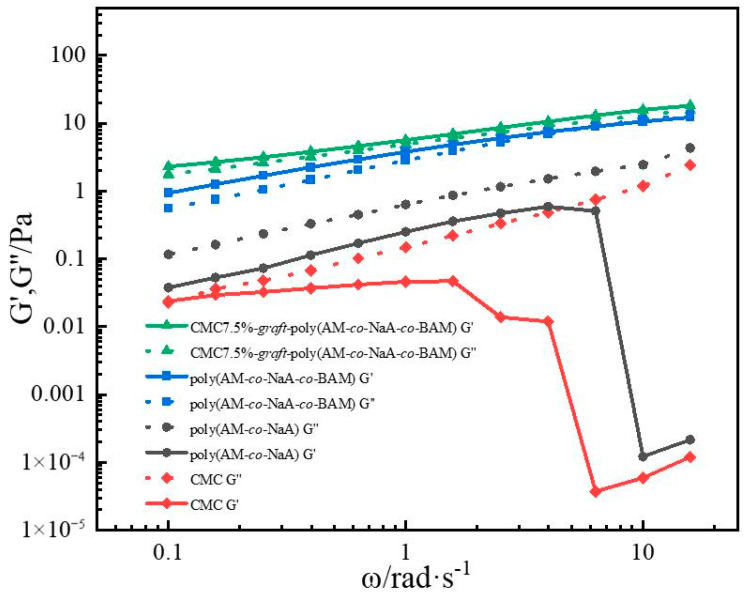
Viscoelastic properties of different copolymers.

**Figure 7:**
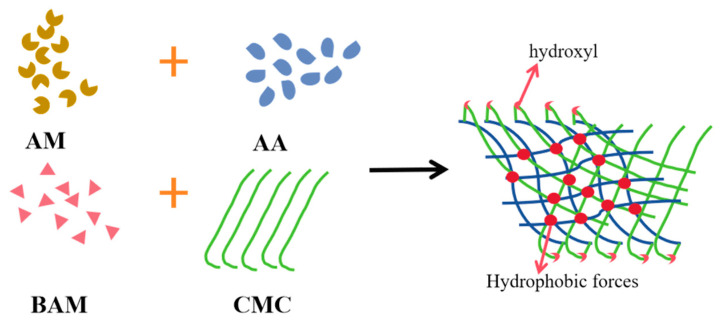
Network structure formed in CMC-*graft*-poly(AM-*co*-NaA-*co*-BAM) solution.

Figure 6 exhibited the rheological curves of the copolymers. Among the four copolymers, CMC7.5%-*graft*-poly(AM-*co*-NaA-*co*-BAM) demonstrated the highest storage modulus (G′) and loss modulus (G″). Elevated elastic modulus (G′) and viscous modulus (G″) are typically attributed to enhanced structural strength and resistance to flow disturbances or deformation, resulting from the formation of particle clusters or flocculates with strong inter-particle bonding. It is because CMC grafting and dual-network design can construct a three-dimensional skeleton with high crosslinking density (Figure 7), coupled with multiple energy dissipation mechanisms (e.g., hydrogen bond reorganization, inter-cluster friction), which collectively confer exceptional structural stability.

#### 2.4.2. Thixotropy Measurement Results

**Figure 8:**
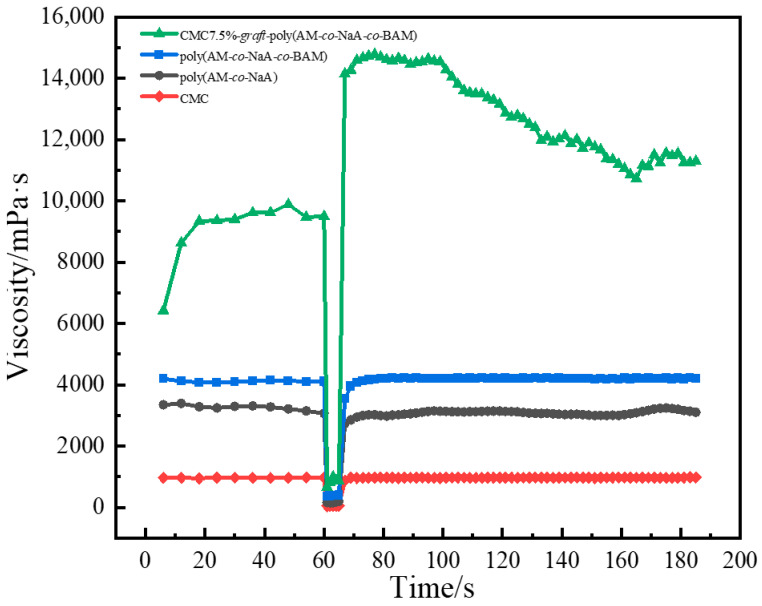
Time-dependent thixotropy test of copolymers.

The thixotropy of the copolymers was evaluated using an Anton Paar rheometer with a three-stage shear protocol: initially, a low shear rate of 1 s^−1^ was applied for 60 s with data recorded every 6 s, followed by a high shear rate of 100 s^−1^ for 5 s with data points documented at 1 s intervals, and finally, the low shear rate of 1 s^−1^ was reapplied for 120 s with observations made every 2 s. As demonstrated in Figure 8, after the high-shear phase, CMC7.5%-*graft*-poly(AM-*co*-NaA-*co*-BAM) rapidly regained its viscosity, exceeding the initial value, whereas poly(AM-*co*-NaA-*co*-BAM), poly(AM-*co*-NaA), and CMC showed considerably lower recovery rates. This superior performance was attributed to the enhanced spatial network structure of CMC7.5%-*graft*-poly(AM-*co*-NaA-*co*-BAM), which facilitated quick reorganization after shear-induced disruption, leading to viscosity recovery that surpassed the original maximum.

### 2.5. Particle Diameter Analysis of Copolymer Solutions

**Figure 9:**
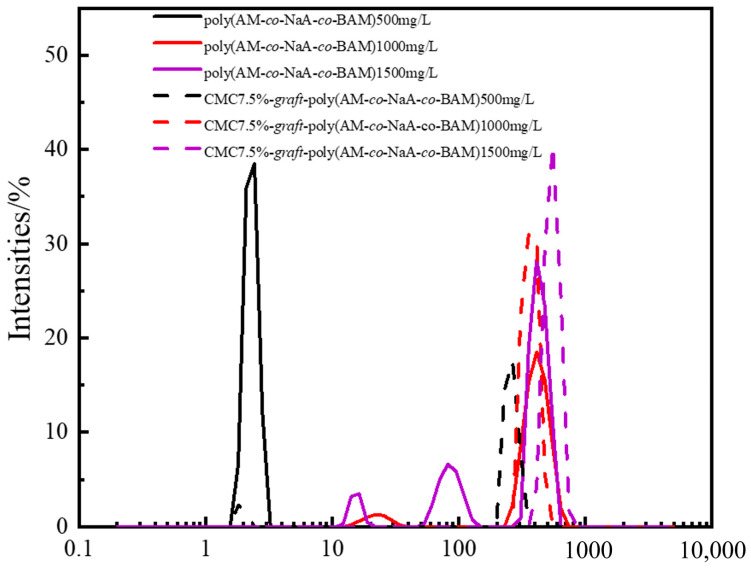
Particle size distribution of copolymers.

The particle size distribution of the copolymers is presented in Figure 9. For poly(AM-*co*-NaA-*co*-BAM), intramolecular association predominated below the critical aggregation concentration (CAC, 500 mg/L), while intermolecular hydrophobic association became dominant above the CAC, leading to a dynamic physically crosslinked network evidenced by a significant increase in particle size and broader size distribution. In contrast, CMC-*graft*-poly(AM-*co*-NaA-*co*-BAM) exhibited a monomodal, continuous distribution across tested concentrations, with larger aggregate sizes than poly(AM-*co*-NaA-*co*-BAM) at equivalent concentrations, indicating a topologically homogeneous crosslinked network at high concentrations. This homogeneity effectively prevents microphase separation—common in bimodal-distributed pure systems—and maintains uniform hydrophobic microdomain dimensions, thereby enhancing the viscosity of the carboxymethyl cellulose-modified amphiphilic polymer more significantly than poly(AM-*co*-NaA-*co*-BAM).

The larger particle size and homogeneous distribution of CMC7.5%-*graft*-poly(AM-*co*-NaA-*co*-BAM) may further contribute to its flame-retardant efficacy by increasing specific surface area, which accelerates water release and vaporization heat absorption (enhancing cooling) and promotes the formation of a denser, continuous protective coating to isolate oxygen and suppress coal-oxygen complex reactions.

Through the aforementioned analysis of the polymer, it was determined that the synthesized carboxymethyl cellulose-modified amphiphilic polymer possesses a branched architecture; in addition to hydrophobic interactions, the grafted carboxymethyl cellulose backbone was found to be rich in hydrogen bonds, thereby endowing it with a significantly enhanced capacity for aggregate formation and enabling the formation of larger aggregates even at lower concentrations [50]. Furthermore, the introduction of hydrophobic groups was observed to facilitate the formation of hydrophobic microdomains, which substantially strengthened both intramolecular and intermolecular association capabilities, consequently resulting in the creation of more active sites and a spatially developed network structure within the polymer system.

### 2.6. Analysis of Hydrogel Adhesion Properties

**Figure 10:**
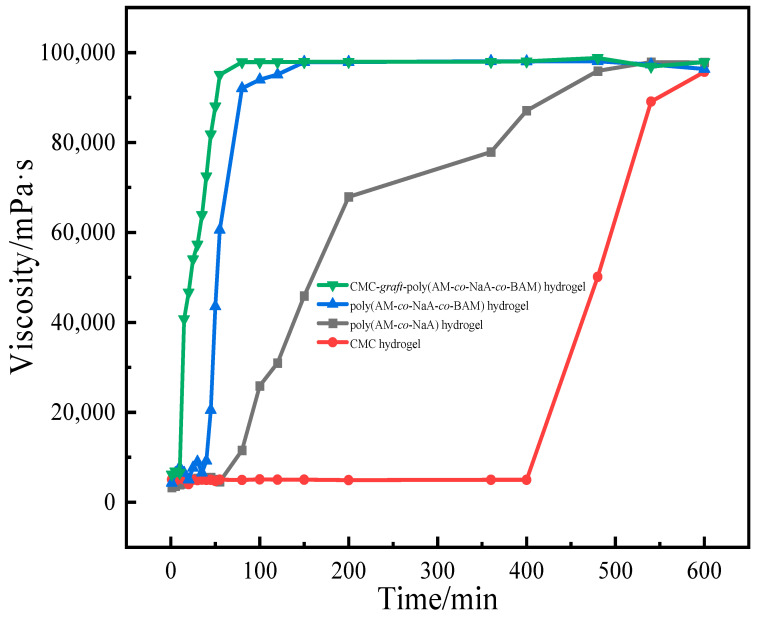
Viscosity-enhancing performance of hydrogels.

Viscosity-enhancing performance of hydrogel was illustrated in Figure 10. CMC-*graft*-poly(AM-*co*-NaA-*co*-BAM) hydrogel exhibited the shortest gelation time and the highest gel strength. The introduction of sodium acrylate and acrylamide significantly increases the density of carboxyl groups (-COO^−^) and amide groups (-CONH_2_), thereby providing abundant coordination cross-linking sites for Al^3+^ ions from aluminum citrate. Meanwhile, the side chain structures derived from acrylamide and N-benzylacrylamide expand the interchain spacing between molecules, leading to the formation of hydrophobic microdomains. This spatial optimization effectively mitigates heterogeneous cross-linking caused by localized enrichment of Al^3+^, thus maintaining uniform and gradual viscosity development throughout the gelation process.

### 2.7. High Temperature Test Analysis

**Figure 11:**
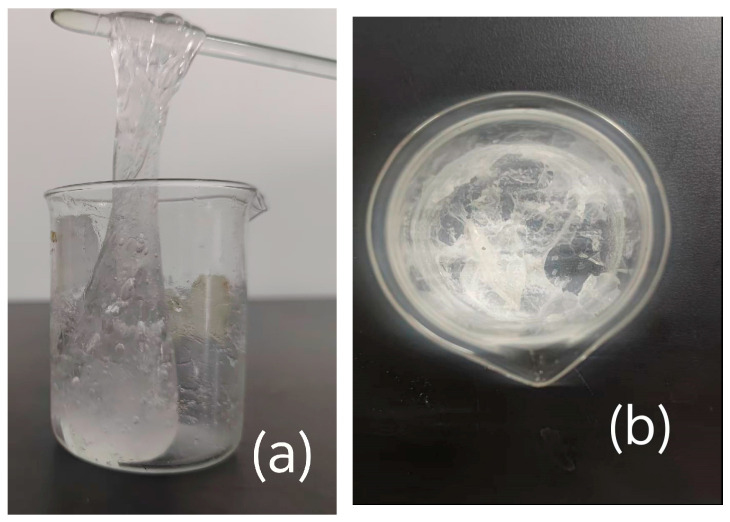
Comparison of the hydrogels before and after high-temperature treatment. (**a**) Hydrogel before high-temperature testing; (**b**) Hydrogel after high-temperature testing.

The water loss behavior of the hydrogels was observed, and the complete dehydration time was recorded as in Table 2. Photographs of the hydrogels before and after high-temperature treatment are presented in Figure 11.

As in Table 2, the dehydration time of CMC-*graft*-poly(AM-*co*-NaA-*co*-BAM) hydrogel was 2.17 times that of pure water at 180 °C. CMC-*graft*-poly(AM-*co*-NaA-*co*-BAM) hydrogel has the best thermal stability, and it can be attributed to the fact that the hydroxyl and carboxymethyl groups on CMC molecular chains establish hydrogen-bonding networks with water molecules. The post-modification introduction of amino groups (BAM) and acrylic acid (NaA) provides additional hydrogen-bonding sites. Furthermore, hydrophobic groups impose steric hindrance effects, which can restrict the permeation of free water.

### 2.8. Rheological Test of Hydrogels

#### 2.8.1. The Shear Resistance Hydrogels

**Figure 12:**
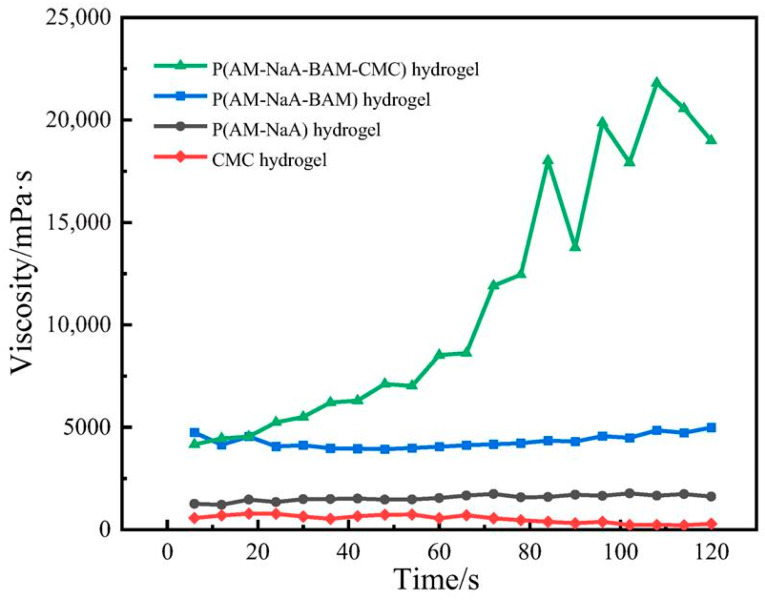
Temporal viscosity evolution of hydrogels under identical shear rate (50 s^−1^).

**Figure 13:**
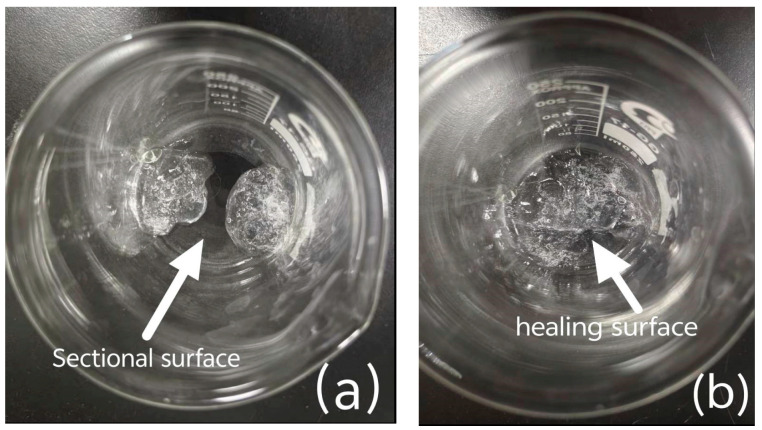
Comparison of CMC-*graft*-poly(AM-*co*-NaA-*co*-BAM) hydrogels before and after cutting. (**a**) Hydrogel after shearing test; (**b**) Hydrogel after self-healing.

Figure 12 showed the shear resistance of the four hydrogels. A constant shear rate of 50 rad/s was selected to measure the viscosity changes in CMC-*graft*-poly(AM-*co*-NaA-*co*-BAM) and CMC hydrogels over 120 s. CMC-*graft*-poly(AM-*co*-NaA-*co*-BAM) exhibited the highest initial viscosity, which enabled rapid adhesion to coal fracture surfaces to mitigate fluid displacement risks from gas/water flow, whereas its dynamic bond reorganization mechanism repairs localized network damage under external stresses (e.g., crack propagation in coal seams, mining-induced vibrations), maintaining structural integrity of the sealing barrier. The schematic diagram illustrating the hydrogel before and after self-healing is presented in Figure 13.

#### 2.8.2. Viscoelasticity Test of Hydrogels

**Figure 14:**
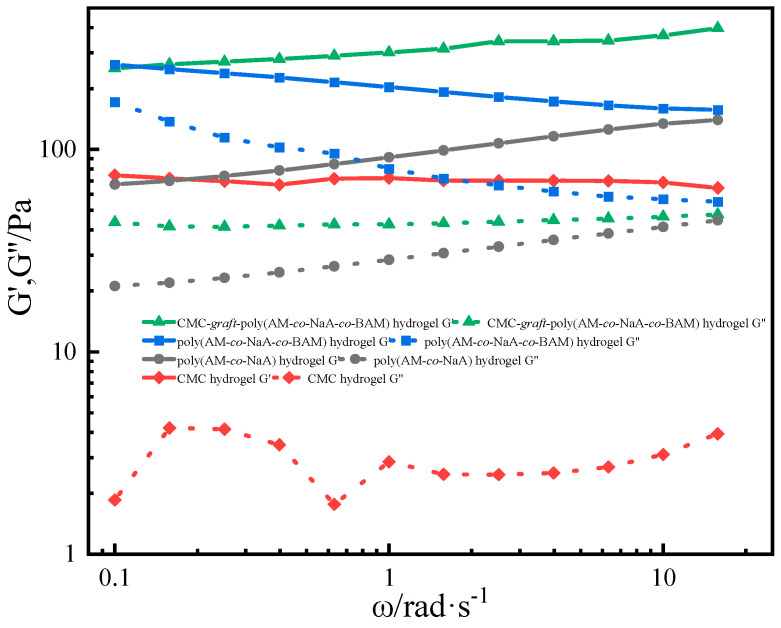
Rheological curves of different hydrogels.

Figure 14 presented the rheological curves of the hydrogels. The storage modulus (G′) quantifies the material’s elasticity, while the loss modulus (G″) characterizes its viscous behavior. At high scanning frequencies, the storage moduli of all four hydrogels exceed their respective loss moduli, demonstrating their predominantly elastic nature over viscous properties. Among the four hydrogel systems, CMC-*graft*-poly(AM-*co*-NaA-*co*-BAM) hydrogel exhibits the highest storage modulus. This difference originates from the fact that, unlike the other three hydrogels, CMC-*graft*-poly(AM-*co*-NaA-*co*-BAM) hydrogel incorporates natural polymer CMC grafted onto the polymer backbone. This modification creates a three-dimensional network structure with the original polymer, while the secondary network further reinforces the architecture through chain entanglement and intermolecular hydrogen bonding with the primary network. In conclusion, CMC-*graft*-poly(AM-*co*-NaA-*co*-BAM) hydrogel demonstrates enhanced elastic properties.

The excellent stability and viscoelasticity of the material are attributed to the crosslinking interaction between Al^3+^ ions and specific functional groups within the polymer. Strong coordination bonds are formed between Al^3+^ and the carboxylate groups (-COO^−^) on carboxymethyl cellulose (CMC) and sodium acrylate (NaA) units, establishing a stable ionic crosslinked network that serves as the primary framework. Simultaneously, weak coordination occurs with the amide groups (-CONH_2_), imparting dynamic reversibility to the network. This dual crosslinking mechanism enhances mechanical strength through rigid ionic bonds while facilitating energy dissipation via dynamic bonds, which is directly reflected in its high storage modulus (G′) and loss modulus (G″).

### 2.9. Simultaneous Thermal Analyzer (STA) Analysis of Hydrogels

**Figure 15:**
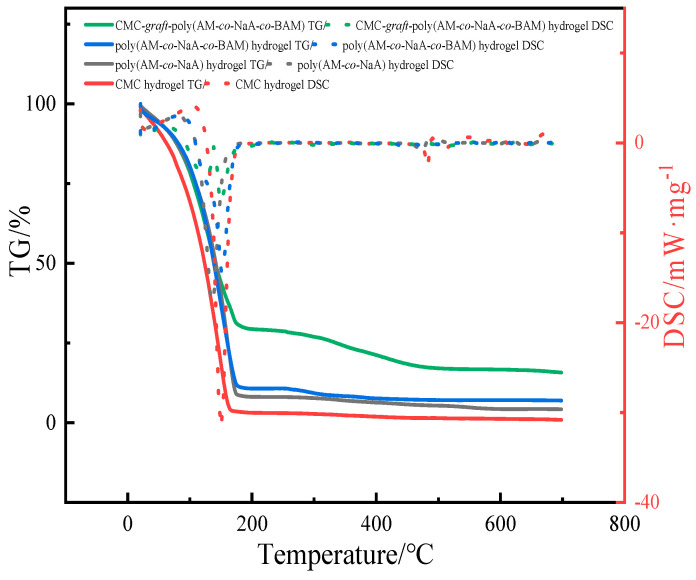
Thermogravimetric (TG) curves and heat flow curves of the four hydrogels.

As shown in Figure 15, the weight loss of CMC-*graft*-poly(AM-*co*-NaA-*co*-BAM) hydrogel exhibited a significantly slower decline compared to the other hydrogels as temperature increases. While all hydrogels undergo rapid mass loss around 180 °C, the mass retention of CMC-*graft*-poly(AM-*co*-NaA-*co*-BAM) hydrogel stabilized near 30%, whereas the others continued to decrease sharply to 10%. Furthermore, the delayed thermal response and enhanced water retention properties can be interpreted through analysis of the characteristic changes in the endothermic peaks associated with water evaporation in the DSC curves. The DSC curves demonstrate that the endothermic peak of the CMC-*graft*-poly(AM-*co*-NaA-*co*-BAM) hydrogel appears in a higher temperature range with a broader shape, indicating that greater energy input and longer duration are required for water evaporation, which directly confirms its delayed thermal response properties. Simultaneously, the larger area of the endothermic peak suggests that a greater amount of bound water needs to be evaporated, verifying its enhanced water retention capacity. This thermal behavior is attributed to the dense hydrogen bond network formed by the carboxymethyl cellulose skeleton and hydrophobic microdomains within the hydrogel, which strongly confines water molecules, significantly elevates the boiling point of water, and slows the evaporation kinetics, thereby maintaining a cooling suppression effect for an extended duration at high temperatures and effectively retarding the coal-oxygen complex reaction.

### 2.10. Analysis of Fire Prevention and Extinguishing Test

**Figure 16:**
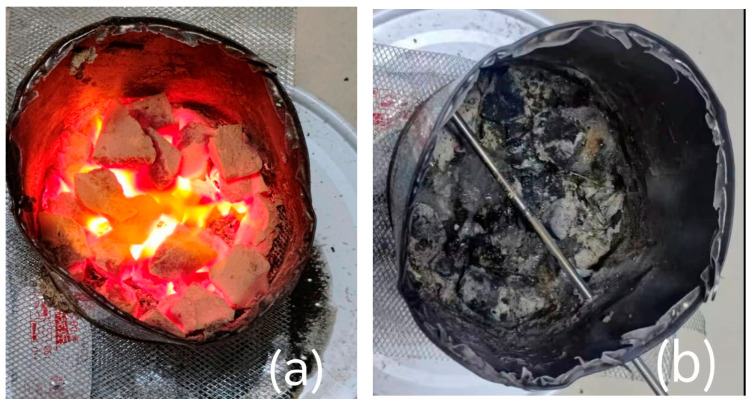
Comparison of coal block before and after the fire-extinguishing experiment. (**a**) Coal block before fire extinguishing with hydrogel; (**b**) Coal block after fire extinguishing with hydrogel.

Figure 16 presents a comparative illustration of the coal block before and after fire extinguishing treatment using the hydrogels.

**Figure 17:**
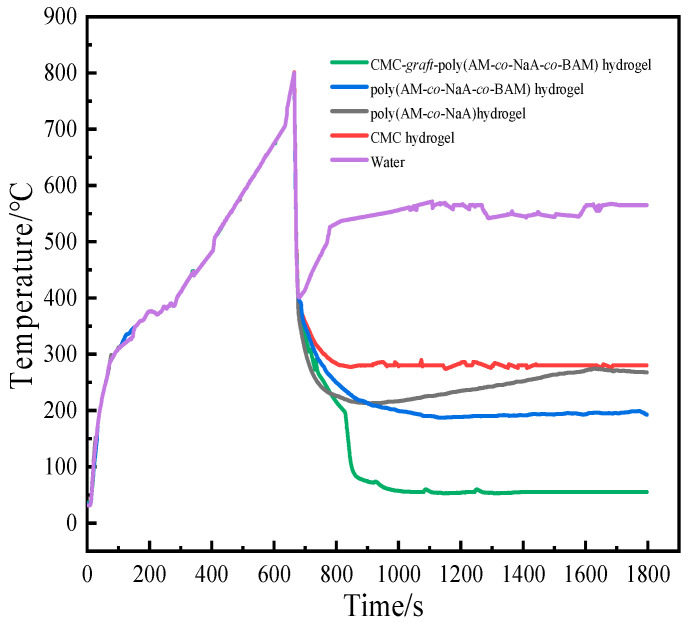
Fire-extinguishing effects of different hydrogels and water.

Significant differences in cooling effects on glowing charcoal were observed among the four hydrogels and pure water in the fire resistance test shown in Figure 17. The charcoal temperature was rapidly reduced from 800 °C to 400 °C within 20 s by all tested samples; however, their performances markedly diverged below 400 °C. Continuous temperature reduction until stabilization at room level without re-ignition was achieved by the CMC-*graft*-poly(AM-*co*-NaA-*co*-BAM) hydrogel, whereas the temperature was only maintained between 150 and 300 °C by the poly(AM-*co*-NaA-*co*-BAM), poly(AM-*co*-NaA), and CMC hydrogels, failing to achieve further cooling and exhibiting clear re-ignition risks. In contrast, although initial cooling effectiveness was shown by pure water, the charcoal temperature rebounded quickly after dropping to 400 °C, resulting in re-ignition and indicating an inability to provide sustained fire isolation and deep cooling. The superior performance of the CMC-*graft*-poly(AM-*co*-NaA-*co*-BAM) hydrogel was attributed to its multi-mechanism synergy: substantial heat was absorbed for rapid cooling through phase change and vaporization of internal water upon contact with the high-temperature source; excellent water retention capacity was provided by its three-dimensional network structure; a dense coating layer was formed on the coal surface, physically isolating oxygen and interrupting the combustion chain; free-radical chain reactions were chemically suppressed by functional groups such as carboxyl and amide; and long-term fire prevention integrity was maintained by the dynamic self-healing property based on hydrophobic microdomains, ensuring autonomous repair of the sealing layer after damage.

## 3. Conclusions

(1) The carboxymethyl cellulose-modified amphiphilic polymer CMC-*graft*-poly(AM-*co*-NaA-*co*-BAM) was synthesized and the successful grafting of sodium carboxymethyl cellulose (CMC) with the hydrophobic monomer (N-benzylacrylamide) and the hydrophobic modification of partially hydrolyzed polyacrylamide were confirmed by infrared spectroscopy and relative molecular mass analysis, which enabled the formation of larger aggregates even at low polymer concentrations; meanwhile, the presence of abundant hydroxyl groups on the polymer chains, combined with the introduction of hydrophobic monomers leading to the formation of hydrophobic microdomains, resulted in strong intramolecular and intermolecular associative capabilities, thereby endowing the carboxymethyl cellulose-modified amphiphilic polymer with superior performance in temperature resistance, salinity tolerance, viscoelasticity, and thixotropy compared to the control group.
(2) Following polymer modification, abundant cross-linking sites were introduced, leading to accelerated gelation and improved mechanical strength. The hydrophobic microdomains, formed via aggregation of hydrophobic groups, acted as physical cross-linking points that interconnected polymer chains into a three-dimensional network. This structure effectively dispersed and dissipated mechanical energy, significantly enhancing the hydrogel’s elastic modulus. Moreover, the dynamic and reversible nature of hydrophobic interactions enabled spontaneous reassociation and reformation of physical cross-links upon fracture surface contact, resulting in autonomous self-healing. The CMC-*graft*-poly(AM-*co*-NaA-*co*-BAM)/AlCit hydrogel exhibited superior water retention and stability compared to the other three hydrogels. Fire suppression tests further demonstrated that incorporating carboxymethyl cellulose-modified amphiphilic polymer hydrogel significantly reduced ignition source temperature and prevented coal re-ignition.

## 4. Materials and Methods

### 4.1. Materials

Acrylamide (AM, AR), sodium dodecyl sulfate (SDS, CP), N-benzyl acrylamide (BAM, AR), and sodium carboxymethyl cellulose (CMC, AR) were purchased from Shanghai, China of Shanghai Titan Technology Co., Ltd. potassium persulfate (KPS, AR) was supplied by Tianjin, China of Tianjin Hengxing Chemical Reagent Co., Ltd. sodium bisulfite (SHS, AR) was obtained from Tianjin, China of Tianjin Jinbei Fine Chemical Co., Ltd. High-purity nitrogen gas (N_2_, AR) was sourced from Anqiu, China of Anqiu Heng’an Gas Factory. Anhydrous ethanol was divided into three batches: purchased from Tianjin, China of Tianjin Hengxing Chemical Reagent Co., Ltd., Laiyang City Kinder Chemical Co., Ltd., and laboratory-distilled in-house. All experiments utilized distilled water prepared in the laboratory.

### 4.2. Synthesis of Cellulose-Modified Amphiphilic Copolymers

**Figure 18:**
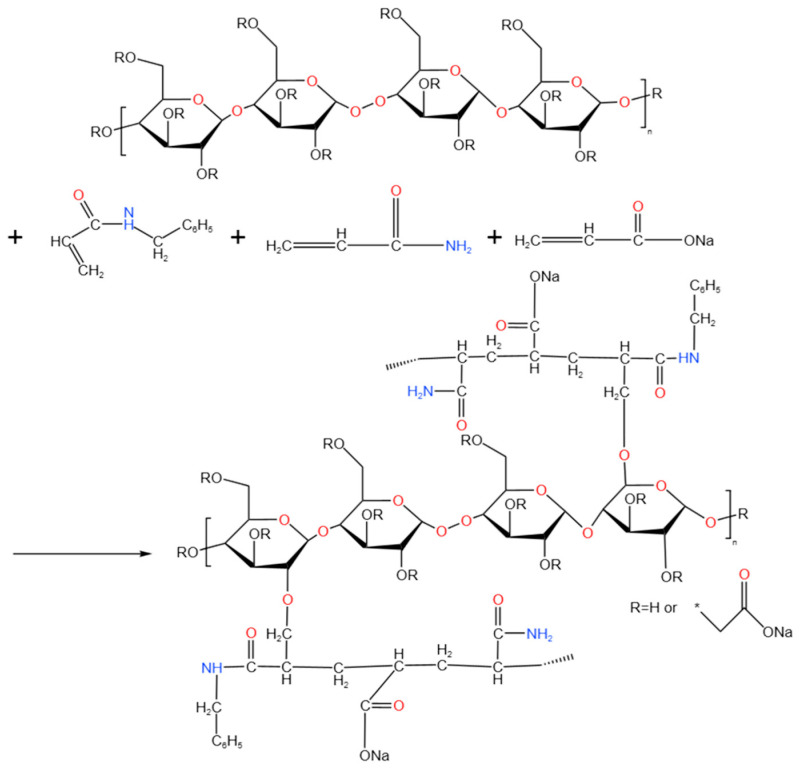
Synthesis reaction mechanism of CMC7.5%-*graft*-poly(AM-*co*-NaA-*co*-BAM).

Carboxymethyl cellulose powder (2.5–10 wt% of total monomer mass) was dissolved in 30 mL distilled water and stirred overnight; the solution was deoxygenated by purging with nitrogen gas (N_2_) for 20 min and then transferred to a three-necked flask. Sodium acrylate (NaA) solution was prepared by dissolving acrylic acid (AA) in 6 N NaOH with the pH between 7 and 8. Subsequently 12.434 g of acrylamide (AM), 21.957 g of sodium dodecyl sulfate (SDS), 7.157 g of NaA, and 0.409 g of N-benzylacrylamide (BAM) were sequentially added to the reaction system. The mixture was placed in water bath at 60 °C. The polymerization was initiated using a redox system comprising potassium persulfate (KPS) and sodium bisulfite (SHS), added at 0.5 wt% relative to the total monomer mass. The reaction proceeded for 8 h under N_2_ atmosphere. The product was fragmented into small pieces and washed three times with anhydrous ethanol and then dried at 45 °C for 48 h. The reaction mechanism of carboxymethyl cellulose-modified amphiphilic polymer is illustrated in Figure 18, respectively. poly(AM-*co*-NaA) and poly(AM-*co*-NaA-*co*-BAM) were also synthesized by the similar method [51,52]. All experiments were repeated three times (as shown in Table 3), thereby providing additional support for the original experimental results and enhancing their persuasiveness.

### 4.3. Viscosity Measurement of Copolymer Solutions

Copolymer thickening capacity was determined using an NDJ-5S rotational viscometer (Shanghai Jitai Electronics Technology Co., Ltd.) from Shanghai of China; temperature resistance performance was quantified at 30~55 °C; and the impact of NaCl and CaCl_2_ on polymer viscosity was systematically tested at different salt concentrations.

### 4.4. Polymer Characterization

#### 4.4.1. FT-IR Spectroscopy

Measurements were performed using a Fourier transform infrared (FT-IR) spectrometer NICOLET 380 (Beijing Edgehawk International Technology Co., Ltd.) from Beijing of China while the scanning range was 4000~300 cm^−1^.

#### 4.4.2. Molecular Weight Determination

The molecular weight of HPAM (partially hydrolyzed polyacrylamide) was calculated via the Mark-Houwink equation. The equation adopted in this study was: *M* = 802[*η*]^1.25^, where [*η*] is the intrinsic viscosity of HPAM measured according to GB/T 12005.1-1989: Determination for Limiting Viscosity Number of Polyacrylamide. The intrinsic viscosity was determined by the dilution method using a conventional Ubbelohde viscometer. Viscosity measurements were conducted in 1.00 mol/L NaCl solution at 30 °C. The initial polymer concentration was 200 mg/L.

In CMC-*graft*-poly(AM-*co*-NaA-*co*-BAM), the molar fraction of hydrophobic monomers is 1%, methyl-β-cyclodextrin was utilized to shield hydrophobic groups and prevent their association [53,54]. CMC-*graft*-poly(AM-*co*-NaA-*co*-BAM) is approximately regarded as HPAM, and their molecular weights can also be calculated by the Mark-Houwink equation as described above under these conditions [52].

### 4.5. Rheological Measurements of Copolymers

The rheological properties were performed using a rotary rheometer from Anton Paar. The linear dynamic viscoelasticity of the CMC-*graft*-poly(AM-*co*-NaA-*co*-BAM) and other copolymers was measured in the compression mode at 25 °C.

The thixotropy of copolymers was tested using an Anton Paar rheometer. Initially, a low shear rate of 1 s^−1^ was applied to the polymer samples for 60 s, with data points recorded at 6 s intervals. Subsequently, a high shear rate of 100 s^−1^ was imposed on the copolymers for 5 s, and data points were documented at 1 s intervals. Finally, a low shear rate of 1 s^−1^ was reapplied to the polymer samples, with observation conducted for 120 s and data points recorded at 2 s intervals.

### 4.6. Copolymer Particle Size Distribution Test

The particle size distribution of copolymer solutions was evaluated using a Malvern laser particle size analyzer (Malvern Instruments Ltd., Malvern, UK), with a measurement range of 0.1–5000 nm being specified for hydrodynamic diameter analysis.

### 4.7. Synthesis and Performance of Hydrogels

**Figure 19:**
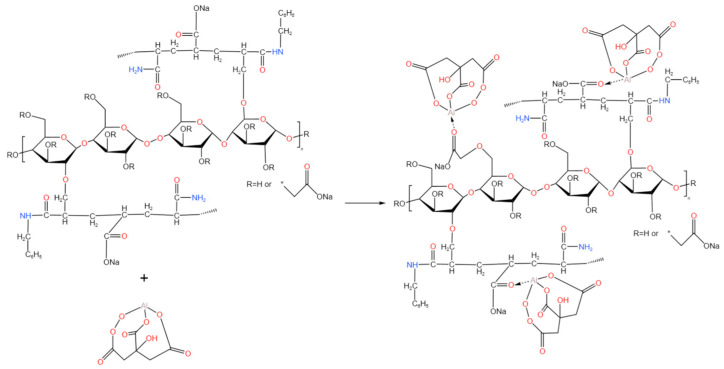
Preparation mechanism for cellulose-modified amphiphilic polymer hydrogel).

The CMC-*graft*-poly(AM-*co*-NaA-*co*-BAM) copolymer was crosslinked with aluminum citrate (AlCit) via an ionic coordination mechanism. Specifically, a 20,000 mg/L AlCit aqueous solution was introduced into a 20,000 mg/L aqueous solution of the copolymer under continuous magnetic stirring at room temperature (approximately 25 °C) for 30 min to ensure homogeneous mixing and complete complexation. The crosslinking mechanism of the resulting hydrogel and the schematic diagram for the synthesis of carboxymethyl cellulose-modified amphiphilic polymer hydrogel are illustrated in Figure 19.

### 4.8. Water Loss Behavior at Elevated Temperature (180 °C)

Water loss behavior of hydrogels was evaluated by simultaneously placing three 50 mL beakers containing the prepared carboxymethyl cellulose-modified amphiphilic polymer hydrogel, carboxymethyl cellulose (CMC) hydrogel, and deionized water (control) into a preheated drying oven at 180 °C, immediately sealing the chamber, and recording the complete moisture evaporation time for each sample.

### 4.9. Rheological Measurements of Hydrogels

#### 4.9.1. Shear Resistance Testing of Hydrogels

This study employed an Anton Paar MCR series rotational rheometer equipped with RheoCompass software (1.35.1394) to characterize the shear resistance properties of carboxymethyl cellulose-modified amphiphilic polymer hydrogels and carboxymethyl cellulose (CMC) hydrogels, specifically analyzing the time-dependent viscosity variation under a constant shear rate of 50 s^−1^ while recording the viscosity values.

#### 4.9.2. Viscoelasticity Testing of Hydrogels

The rheological properties were performed using a rotary rheometer from Anton Paar. The linear dynamic viscoelasticity of the CMC-*graft*-poly(AM-*co*-NaA-*co*-BAM) hydrogel and other hydrogels was measured in the compression mode at 25 °C.

### 4.10. Simultaneous Thermal Analysis

Simultaneous thermal analysis (STA) tests were performed on carboxymethyl cellulose-modified amphiphilic polymer hydrogels and carboxymethyl cellulose (CMC) hydrogels using a NETZSCH STA 449 F3 analyzer (Naichi Scientific Instruments Trading Co., Ltd., Shanghai, China), integrating thermogravimetric analysis (TGA) and differential scanning calorimetry (DSC) under controlled temperature conditions: 25–700 °C, heating rate: 10 °C/min. Mass changes were monitored via a high-sensitivity balance, while DSC detected heat flow differences, enabling precise quantification of thermal effects, including phase transitions, melting, and crystallization.

### 4.11. Fire-Extinguishing Experiments

**Figure 20:**
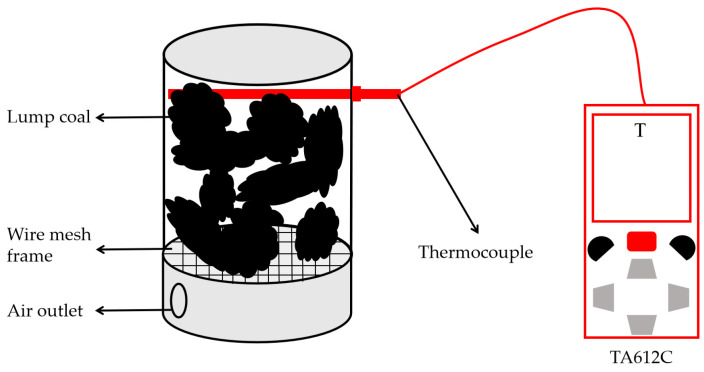
Experimental setup of fire extinguishing apparatus.

The fire-extinguishing performance of the hydrogels was evaluated using a custom-designed experimental apparatus; coal blocks were ignited to simulate spontaneous coal combustion, with water or gel injected into the apparatus upon reaching 800 °C coal temperature while monitoring temperature using a Tansi TA612C thermocouple (Suzhou Teans Electronics Industrial Co., Ltd., Suzhou, China) at 1 s intervals until stabilization, followed by comparison of fire suppression effects among water and hydrogels, with the experimental configuration detailed in Figure 20.

## Figures and Tables

**Table 1:** The characterization of relative molecular weight.

Copolymers	*η_r_*	[*η*] (mL/g)	*Mv* (g/mol)	*M*
CMC2.5%-*graft*-poly(AM-*co*-NaA-*co*-BAM)	1.33	598.40	2959.64	2.37 × 10^6^
CMC5%-*graft*-poly(AM-*co*-NaA-*co*-BAM)	1.31	555.81	2698.66	2.16 × 10^6^
CMC7.5%-*graft*-poly(AM-*co*-NaA-*co*-BAM)	1.38	672.20	3422.73	2.75 × 10^6^
CMC10%-*graft*-poly(AM-*co*-NaA-*co*-BAM)	1.29	544.20	2628.44	2.11 × 10^6^

**Table 2:** The water loss behavior of hydrogels at 180 °C.

Sample	CMC-*graft*-poly(AM-*co*-NaA-*co*-BAM) Hydrogel	poly(AM-*co*-NaA-*co*-BAM) Hydrogel	poly(AM-*co*-NaA) Hydrogel	CMC Hydrogel	Water
Complete dehydration time/min	105.80∽107.00	88.50∽91.50	82.80∽84.30	70.20∽71.90	48.70∽50.30
Ratio to water evaporation time	2.15 ± 0.04	1.81 ± 0.07	1.68 ± 0.04	1.43 ± 0.04	1.00

**Table 3:** The feeding composition of copolymers.

Samples	Feed Ratio of Reactants (wt%)				Initiator Ratio * (wt%)	Total Mass (g)	Yield (%)
	AM	NaA	BAM	CMC			
CMC7.5%-*graft*-poly(AM-*co*-NaA-*co*-BAM)1	62.28	35.85	1.87	7.50	0.75	20.00	82.62
CMC7.5%-*graft*-poly(AM-*co*-NaA-*co*-BAM)2	62.28	35.85	1.87	7.50	0.75	20.00	81.41
CMC7.5%-*graft*-poly(AM-*co*-NaA-*co*-BAM)3	62.28	35.85	1.87	7.50	0.75	20.00	82.20
poly(AM-*co*-NaA-*co*-BAM)1	62.28	35.85	1.87	0.00	0.75	20.00	87.64
poly(AM-*co*-NaA-*co*-BAM)2	62.28	35.85	1.87	0.00	0.75	20.00	89.45
poly(AM-*co*-NaA-*co*-BAM)3	62.28	35.85	1.87	0.00	0.75	20.00	86.26
poly(AM-*co*-NaA)1	62.28	35.85	1.87	0.00	0.75	20.00	91.13
poly(AM-*co*-NaA)2	62.28	35.85	1.87	0.00	0.75	20.00	90.44
poly(AM-*co*-NaA)3	62.28	35.85	1.87	0.00	0.75	20.00	90.18

* The mass fraction of KPS and SHS relative to the total monomer mass.

## Data Availability

The original contributions presented in this study are included in the article. Further inquiries can be directed to the corresponding author.
